# ACTIVE IMMUNIZATION AGAINST MYOSTATIN AND ACTIVIN A IMPROVES SKELETAL MUSCLE STRENGTH DESPITE GH/IGF-1 DEFICIENCY

**DOI:** 10.1093/geroni/igae098.3631

**Published:** 2024-12-31

**Authors:** Mishfak Abdullah Mohamed Mansoor, Sarah Ashiqueali, Md Tanjim Alam, Amanda Lasseter, Brett Thibodeaux, Michal Masternak

**Affiliations:** University of Central Florida, Orlando, Florida, United States; Mayo Clinic, Rochester, Minnesota, United States; University of Central Florida, Orlando, Florida, United States; Vaxxinity, Merritt Island, Florida, United States; Vaxxinity, Merritt Island, Florida, United States; University of Central Florida College of Medicine, Orlando, Florida, United States

## Abstract

Muscle wasting is a significant hurdle in patients due to immobilization, prolonged bed rest, and injury. In particular, the older population develops sarcopenia, characterized by progressive loss of muscle strength due to natural aging. However, there are no FDA-approved drugs for sarcopenia, and it is critical to explore novel therapeutics. Myostatin inhibition is one such strategy that has had varying degrees of success previously, yet the desired levels of muscle strength have not been achieved. Here, we employed a novel vaccine (V13) that generates anti-myostatin antibodies and another (V07) that generates anti-activin A; myostatin and activin A regulate muscle growth negatively. We administered seven doses of V13, V07, and V35 (generates both anti-myostatin and anti-activin A) to long-living, GH-deficient Ames dwarf mice (df/df) and their normal littermate controls (N/df). By week 24 post-first dose, we saw increased forelimb grip strength in V13, V07, and V35 vaccinated df/df mice and V07 and V35 vaccinated N/df mice. Additionally, almost 40% of the vaccinated df/df mice completed the accelerating rotarod trials, compared to the 20% of placebo-treated counterparts. We observed increased lean body mass and reduced fat mass in vaccinated N/df mice, yet surprisingly there were no changes in lean and fat mass in df/df mice. In summary, our results indicate that while myostatin and activin A inhibition play an intimate role in fat metabolism in the presence of GH/IGF-1, this immunization strategy improves muscle motor performance in GH/IGF-1 deficient mice providing promising treatments in older animals regardless age-associated suppression of GH/IGF-1 axis.

